# Living collections: care and curation at Drosophila stock centres†

**DOI:** 10.1017/bjt.2019.14

**Published:** 2019-09-16

**Authors:** Jenny Bangham

**Affiliations:** * Department of History and Philosophy of Science, University of Cambridge, Free School Lane, CB2 3RH, UK

## Abstract

Biological stock centres collect, care for and distribute living organisms for scientific research. In the 1990s, several of the world’s largest Drosophila (fruit fly) stock centres were closed or threatened with closure. This paper reflects on why this happened, and uses the visibility of these endings to examine how stock centre collections are managed, who maintains them and how they are kept valuable and accessible to biologists. One stock centre came under threat because of challenges in caring for flies and monitoring the integrity of stocks. Another was criticized for keeping too many ‘archival’ stocks, an episode that reveals what it can mean for a living scientific collection to remain ‘relevant’ to a research community. That centre also struggled with the administrative and documentary practices that have proved crucial for sustaining a collection’s meaning, value and availability. All of the stock centres in this story faced challenges of how to pay for care and curation, engaging with a problem that has been discussed by biologists and their funders since the 1940s: what are the best models for stock provision, and how could these models be changed?

In 1997 the ‘Mid-America’ Drosophila fruit fly stock centre lost its federal funding and closed. Based at Bowling Green State University, Ohio, it was one of only a handful of publicly funded stock centres in the world, and one of three threatened with closure during the 1990s, a situation that, some scientists argued, imperilled the future of Drosophila genetics.^1^ Stock centres collect, care for and distribute living organisms for scientific research. They keep alive thousands of collections of yeasts, bacteria, viruses, plasmids, cell cultures, animals and plants, and they maintain the composition of these collections in ways that shape and are shaped by research.^2^ Stock centres devoted to Drosophila were among the earliest such institutions established, and over a century became essential to worldwide networks of geneticists. But although stock centres are crucial components of biological research infrastructure, their expertise, funding and organization are largely invisible, even to their users.

The threat of closure, however, brought the work of Drosophila stock centres to light. The Mid-America Stock Center closed because – as a collections manager at another institution put it – it was not being ‘actively curated’. Four years later, Europe lost its only publicly funded stock centre, at Umeå, Sweden, because it was unable to secure reliable European Union funding. During the same decade, the US National Drosophila Species Stock Center, which maintained several hundred species of Drosophila, faced critical challenges in caring for its stocks and distributing flies to labs, problems that were resolved only when the centre came under new custodianship at Tucson, Arizona. This article uses written sources and interviews to explore what these endings, threatened and real, reveal about the histories of the curation of living research collections. It also contributes to the growing literature on the classificatory work involved in international biological databases, by focusing on dynamics of institutions and on the maintenance practices and affective labour of individual actors.

In defining the term ‘stock’, the *OED* refers to ‘a store or provision to be drawn upon as occasion requires’, the ‘source of a line of descent’, and ‘the trunk or stem of a living tree’.^3^ Drosophila geneticists use the term (and the synonyms ‘strain’ or ‘line’) to refer to an interbreeding population of flies of shared descent, circumscribed by a vial, bottle or cage, which researchers can breed and use for experiments (Figure 1). Fruit flies tend to be tiny (two millimetres long) and unremarkable to look at (brown and grey) and are long-standing companions to humans, most commonly encountered by people in kitchens and pubs.^4^ In laboratory settings they are extensively engaged in reproductive labour to serve human needs: there exist no ethical protocols for their care, they are easy to cook for and feed, they can be sent through the post, and they move (fairly) effortlessly through border controls.^5^ They are animals with specific and well-defined habits and appetites, but are also highly constructed technical objects that over the last century have been systematically altered by scientists in ways that make them both tools for manipulating genetic crosses and objects of inquiry in their own right.^6^

From the 1910s, scientists isolated and bred mutant fruit flies to produce stocks with specific characteristics. From the 1920s, they brought into the lab strains with unusual chromosomal properties, and used them in breeding experiments to control the inheritance of specific traits.^7^ During that decade, too, researchers began using X-rays to deliberately induce mutations in flies, and from the 1960s used ethyl methane sulfonate for similar effects. In the 1970s, the numbers of Drosophila stocks circulating among labs were sharply amplified as researchers began making genetic ‘screens’; that is, collections of stocks (sometimes many thousands), each with different gene insertions. During the 1980s Drosophila researchers generated mutants using techniques classed as ‘transgenic’.^8^ Meanwhile, throughout the century, evolutionary biologists and entomologists have collected species and strains of Drosophila from natural habitats and transformed them into ‘wild-type’ lines to be studied in labs. In these ways, geneticists have refracted *D. melanogaster* and its sister species into hundreds of thousands of distinct kinds of animal.^9^ Thus, whether wild type, mutant or transgenic, the flies of a single stock share a line of descent. A ‘stock’ is a living culture, perpetually maintained, generation after generation, with stable, recognizable genetic and phenotypic features. But it can be expanded and multiplied, replicated and circulated beyond a lab or stock centre, and a stock in any particular collection can potentially be replaced by ‘copies’ cared for elsewhere.

Drosophila stock centres are institutions that define, care for and distribute thousands of distinct stocks. This article makes a first step towards sketching a history of Drosophila stock centres in relation to a century of fruit fly genetics. The picture is complex, in part because ‘stock centre’ is not a clearly defined institutional entity. Since the 1910s, geneticists have freely posted flies between laboratories. Historian Robert Kohler has shown how these exchange practices functioned as part of a tightly controlled moral economy, whereby a few powerful individuals promoted the lab-tolab distribution of flies to share the labour of genetic mapping and keep tabs on who was working on what.^10^ From the 1930s, the dominant Drosophila lab at Caltech in Pasadena maintained nearly six hundred distinct stocks, for which it employed a dedicated curator.^11^ Since then, many labs have employed workers with the identities of ‘stock keeper’, ‘curator of stocks’ or ‘stock custodian’.^12^ ‘Stock centre’ was a phrase used by Drosophila researchers in the 1950s when the Caltech lab became the first to garner funding from the National Science Foundation (NSF) specifically for stocks.^13^ But laboratories with such funding were never the only places with collections. Many genetics labs typically kept (and still keep) hundreds and sometimes thousands of lines, which they customarily share with other researchers for no financial remuneration. Labs might supplement their local collections by ordering lines from stock centres, but some subfields still rely far more on lab-to-lab exchanges (or on freshly caught field collections) than they do on stock centres. The line between ‘stock centre’ and ‘research lab’ is blurred in other ways too: existing major stock centres remain closely connected to thriving research labs, and stock centre staff are often engaged in scientific research projects. Some present-day labs and university departments run their fly facilities partly as commercial enterprises, making and selling transgenic flies to order to mitigate their costs.

The threatened endings to Drosophila stock centres in the 1990s offer a specific moment for exploring the histories of four institutions widely known as ‘stock centres’ and funded as such.^14^ For clarity, and following the practices of fruit fly scientists, I refer to the centres as the Mid-America Stock Center (closed), Umeå (closed), and the Species Stock Center (saved). As a counterpoint I also reflect on the history and present-day practices of today’s principal Drosophila stock centre: at Bloomington, Indiana. The Bloomington Drosophila Stock Center (BDSC) cares for and distributes more than seventy thousand separate stocks, many of which are cultures from earlier collections at Caltech and other places.^15^ Particularly invested in curatorial expertise and labour, and logics of acquisition and loss, these four institutions are case studies for learning about living biological collections, the practices of curation, documentation and funding, and how these can end.

The English word ‘curation’ comes from *curare*, which means ‘to cure’, and today has meanings relating to care, guardianship and management. I explore three aspects of curation at Drosophila stock centres. The first is the necessary ongoing care that maintains the existence of Drosophila stock collections.^16^ It is not yet possible to reliably suspend Drosophila cultures by freezing embryos for future use, so stock keepers must actively tend to stocks in order to maintain their viability.^17^ Drosophila stocks are typically kept in vials containing a jelly-like mixture of yeast, agar and sugar (Figure 1). People caring for stocks typically ‘flip’ flies onto new food every two weeks, discarding old vials containing sticky food, pupae, larvae, and dead flies. The technology of these routine ‘endings’ is the autoclave – a large box-like chamber designed to sterilize laboratory and medical samples and equipment using high-pressure steam.^18^ As I describe in the first section, without the careful, well-regulated feeding and flipping of vials – and the discarding of dead and unwanted organisms – the stock itself may be lost.^19^

In the second section, I turn to the curatorial work that defines the composition of a stock collection, and keeps that collection relevant to its users. The term ‘collection’ is flexible: here, I usually use it to refer to all of the lines kept by a particular institution, but stock centres might also maintain sub-collections with distinctive identities.^20^ Some of the collections I discuss have been maintained for many decades – the Species Stock Center has some stocks first collected in the 1930s. Methods shift, disciplines die, knowledge changes: keeping stocks alive takes time, labour and money, and laboratories and stock centres routinely assess which stocks they can afford (and need) to keep. The responsibility for acquiring and de-accessioning stocks (in some cases whole subcollections) falls on collections managers, who work to maintain the relevance of collections to users. For a living biological stock centre to thrive it must maintain a vigilant commitment to de-accessioning stocks. Culling and de-accessioning practices are necessary to keep these living collections scientifically useful.

A related subset of curation practices is the continual labour of documentation that maintains a collection’s meaning, value and availability.^21^ For the vast majority of stocks, one mutant or transgenic fly looks very much like another. A stock is useless unless it is labelled correctly, and unless a researcher can readily access information about its genotype, its provenance and its use in past experiments.^22^ Collections managers negotiate naming standards, respond to changes in nomenclature and update databases. For over a century geneticists have engaged in large-scale mapping projects, making them heavily dependent on the shared negotiation of names and nomenclatures.^23^ From the 1920s, distinct research communities negotiated and defined names and symbols using newsletters and mutant catalogues; in the 1990s these paper technologies were (partially) replaced by model-organism-specific databases.^24^ Today, tight collaborations between stock centres and model-organism databases are crucial to biology.^25^ The third section looks in detail at how these relationships have worked (or not) in practice, and how – in the case of Drosophila – they resulted in the flourishing of one stock collection and the end of another.

The paper ends with another crucial aspect of stock centres’ continuity: their ability to secure funding. If curation depends on specialist labour and expertise, who pays for it? How do scientific communities decide on and coordinate the maintenance of collections? How have researchers agreed (or not) on the best models for stock provision, and how have those models changed?

## Care and feeding

During the 1990s researchers made a number of private and public complaints about the integrity of stocks distributed by the US National Drosophila Species Stock Center. One influential biologist and significant donor of flies to the centre complained of stockkeeping ‘disasters’.^26^ With over 1,500 stocks representing three hundred Drosophila species on its books, the centre had to contend with special challenges of fruit fly care.^27^ Most Drosophila labs and stock centres deal with just a single species, *D. melanogaster*, the protocols for which have changed little in eighty years.^28^ *D. melanogaster* stocks are typically kept in vials two centimetres in diameter and ten high, plugged with cotton wool. The bottom section of the vials contains a firm, jelly-like mixture of agar, yeast, anti-fungal chemicals, cornmeal and sugar; the flies live on top. Adult flies lay eggs on the food’s surface, and larvae hatch and burrow, eventually pupating on the interior sides of the vials. Adult *D. melanogaster* live for about a month; before that a vial of flies can become overpopulated, and the food sticky and wet; after two to three months its entire contents dry out. The *D. melanogaster* life cycle at 25° (a temperature commonly used in genetics laboratories) is about ten days. But other Drosophila species, which have been used extensively in evolutionary and ecological research for over a century, have other appetites and lifestyles. To cater for the hundreds of species from different ecological environments, the Species Stock Center lists on its website eleven different recipes – from the ‘Applesauce Food recipe’, to ‘Saguaro Potato recipe’ to ‘Senita Cactus Requirement’. The Species Stock Center collection was first established in the 1940s (some original stocks remain in the collection today), and over the years managers have altered its recipes. Today the ‘list of ingredients’ on the Species Stock Center website includes Opuntia cactus powder, Betty Crocker® instant mashed potato and Kellogg’s breakfast cereal. One collections manager explained how capricious some species could be: ‘there are some that will just stop laying eggs if … the temperature, pressure … you know there are odd weather things that they’re very picky about’.^29^ Fruit fly ‘husbandry’ (the Species Stock Center uses this agriculturally inflected term on its website) can be laborious. When flies are ‘picky’ about their diet, temperature and humidity, different stocks need different methods of care to be kept alive.

**Figure 2:**
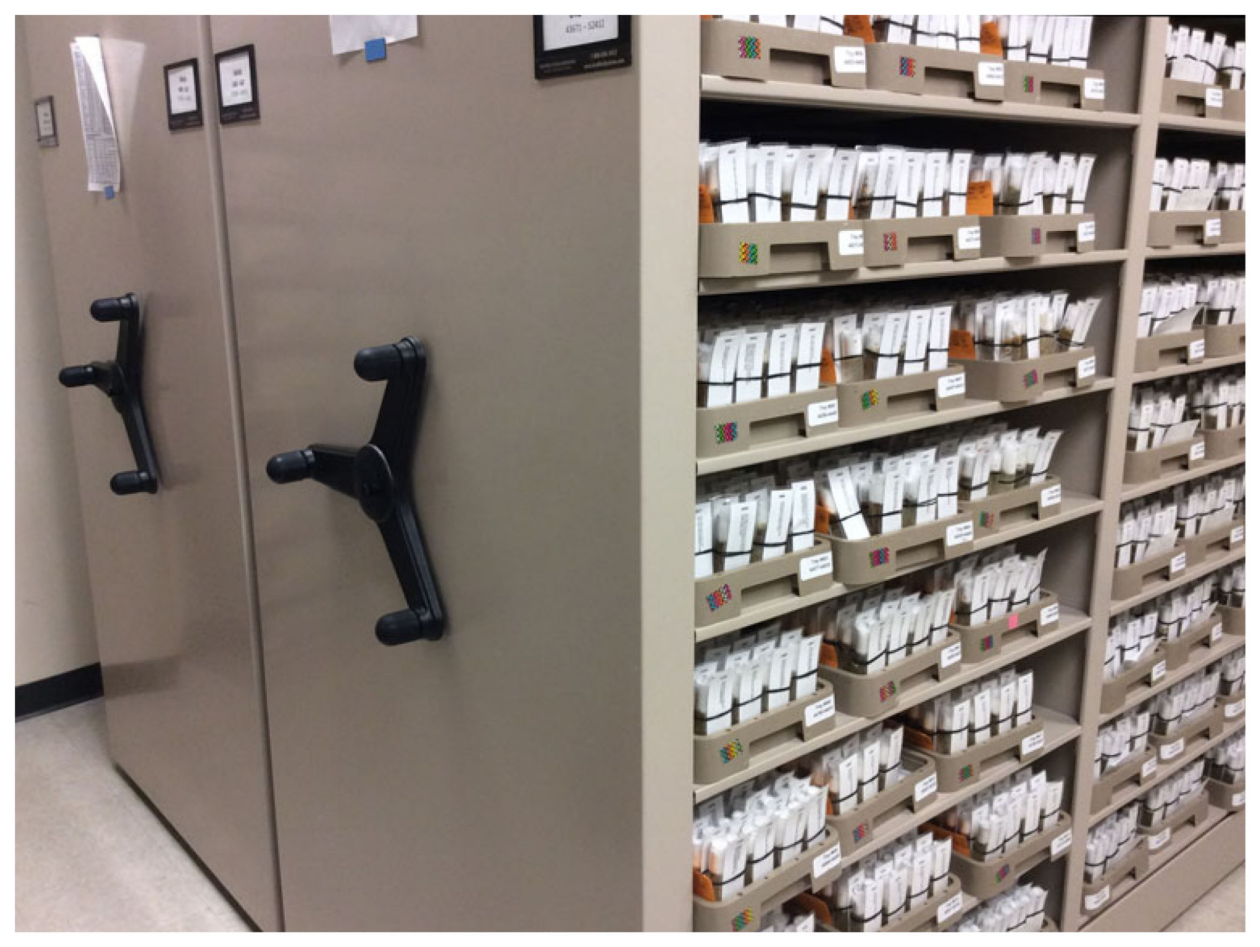
The main storage room for the BDSC Drosophila collection, with moveable library shelving. One collections manager remarked that some of the stock keepers chose to make their stocks look ‘more distinctive’ by decorating their trays with coloured labels and tape. Photograph by the author.

While the Species Stock Center cares for a wide variety of Drosophila, the BDSC looks after thousands of strains of *D. melanogaster*. The BDSC is on the fifth floor of one of several buildings that house the Department of Biology at the University of Indiana in the small city of Bloomington. It keeps its collection of at 22 °C, which slightly extends the fly’s life cycle to about two weeks – far more commensurate with a human workforce than the ten-day cycle used by most labs. At Bloomington each stock is kept in two or more replicate glass vials, bundled together with elastic bands and marked with large, removable, laminated labels (Figure 2). Bundles are kept in plastic trays lined with cheesecloth treated with the mite repellent benzyl benzoate. Each stock has a replicate backup in the basement of the building, although these are slated to move off the main campus to a building that formerly housed the university cyclotron.

Altogether the BDSC holds about 300,000 active cultures, the day-to-day care of which falls to fifty-five or so stock keepers, who ‘flip’ the vials onto new food every two weeks. A cook prepares fly food in a dedicated kitchen, equipped with restaurantgrade stainless steel cooking utensils. *D. melanogaster* cultivation is relatively flexible – stock keepers are free to flip their vials at any time, day or night (one person I spoke to routinely arrived at 4 a.m. and left by 11 a.m.). Most of the stock keepers work part-time (the team of fifty-five corresponds to twenty-two ‘full-time equivalents’), and most are women.^30^ One former (female) stock keeper remarked that the ‘best workers’ were mothers, who typically arrived early, carried out their stock-keeping work, and then left to take care of their children – the rhythms of fly keeping making it possible to dovetail two kinds of reproductive labour. Each stock keeper looks after a stable subset of flies and has their own trays of stocks, and full-time workers flip on average eight or nine trays of vials a day, alongside other tasks such as setting up orders, packaging stocks into cardboard boxes to be sent out, training new stock keepers, making antibiotic-treated and anti-fungal-treated food and monitoring mite deterrents. Although there is apparently little scope for creativity in the flipping of flies – in part because the *D. melanogaster* kept by the centre have been made relatively uniform with respect to their needs – stock keepers shape their work environments.^31^ Many use pieces of coloured card and tape to make their own stock trays more distinctive, so they can easily locate the ones they need to look after (Figure 2). Stock keepers themselves are subject to practices of care: BDSC managers make sure all stock keepers do a range of work to mitigate repetitive-strain injuries. Although the work there is flexible, the lives of all humans who work at stock centres are shaped to some extent by the proclivities and rhythms of the flies – human workers get used to the smell of yeasty Drosophila vials, and they conform to work patterns that broadly cohere with the rhythms of egg laying and pupation.

Biologists in other fields of research sometimes express surprise that Drosophila stock keeping has not become more fully automated. In fact, at least one institution cultivates stocks by non-human means. The Janelia neurobiological research facility, at the privately funded Howard Hughes Medical Institute near Washington, DC, has a stock facility that uses robots to flip vials.^32^ But the conspicuous presence of a video of the robot on their website testifies that Janelia is unusual. BDSC workers argue that although robots are efficient, it is essential that a trained person check the health of a stock as they flip. This points to an important feature of mutant and transgenic ‘stocks’: their existence and identities are not just constructed through genetic manipulation but also by the routines of those who care for them. Some genetic variants find it hard to survive and reproduce, even in the cushy environment of a laboratory vial. These stocks – which one worker described as ‘frail and pampered creatures’ – need specific kinds of monitoring and care.^33^ Indeed, at the BDSC, the most experienced stock keepers on the team look after the most vulnerable flies. One stock keeper, who has worked at Bloomington for several decades, described her stocks as objects of ongoing maintenance and enduring concern: she commented that when she retires, ‘I’ll still worry about them.’^34^ So although the routines of the BDSC do not affect workers quite as precisely as they shape the rhythms of the flies, the collections engender habits of responsibility and concern.

Institutions vary in how precisely fly care affects the lives of workers. The dovetailing of human stock-keeping labour and Drosophila reproductive labour operates differently at different sites and institutions. At the Species Stock Center – today housed at the Cornell University College of Agriculture and Life Science – funding limitations mean that stock keeping is carried out by twelve undergraduates, each working a few hours a week. This workforce is conveniently flexible but is also prone to leaving campus during national holidays, which poses management challenges. Smaller collections of flies in research labs have the potential to exert far greater control over the lives of stock keepers, who are often PhD and postdoctoral researchers. My own recollections of being a Drosophila postdoc are of strict conformity to the ten-day life cycle of Drosophila (at 25 °C), entailing many lost weekends and late nights. The stocks that we used for experiments were collected and constructed locally and we kept few, if any, backup copies in other labs; losing a collection of stocks would mean losing some lines forever.

Turning to another aspect of ongoing maintenance, the BDSC also has three or four staff responsible for investigating stock contamination and chromosome breakdown. Many Drosophila are genetically complex; mutant constructs can unexpectedly disappear. Lines carrying, for example, ‘attached-X’ chromosomes can sometimes revert to normal X chromosomes, so that a stock no longer has a clearly identifiable phenotype. At the BDSC, those who monitor stocks have doctoral or postdoctoral training and are known colloquially at the centre as ‘the scientists’, a term that distinguishes them from the stock keepers. If a stock recipient thinks that it might not be what is declared on its label, these workers investigate. Their space is equipped with microscopes and magnifying lenses, which they use to check that stocks ‘are what they are meant to be’– work that the BDSC website calls ‘quality control’.^35^ The diagnosis of any single stock can take several weeks: dissecting its genetic constitution is a process that requires workers to carry out controlled genetic crosses and examine offspring to check whether genetic markers segregate as expected. If a genetic construct breaks down, collections managers might replace the stock with its backup, or they might try to rebuild it. Occasionally a stock cannot be rescued and is lost completely.^36^

It was these practices that came under scrutiny at the Species Stock Center during the 1990s. Recipients complained that the flies they had received did not seem to be those they had ordered. One member of the institution’s oversight committee complained that a lack of surveillance made him ‘really fed up’ and want to resign.^37^ The NSF, which funded the centre, became increasingly anxious for it to come under new management, and centre managers themselves were keen to retire.^38^ After a period of considerable uncertainty, the stock centre was moved to Tucson under new custodianship.

The difficulties faced by the Species Stock Center, and the ongoing routines of the BDSC, draw attention to the extensive, and gendered, labour and expertise needed to maintain this scientific infrastructure. Feeding, surveillance and repair all help to stabilize otherwise dynamic, breeding and potentially unruly living organisms. They shore up standards that make experiments and laboratories commensurate. They counter temporal change, maintaining the identity and integrity of strains and genetic constructs that might be decades old. They work against the extinction of particular stocks, and against the dispersal of the collection as a whole. These practices do not only apply to collections – a scientist maintaining a single stock will use similar propagation techniques – but stocks brought together into collections also bring into being standardized routines, specialized labour and professional identities. These are the basis on which are built other kinds of curation, to which I turn next.

## Maintaining relevance

The value of living biological collections depends on their continued relevance to the communities that they serve. Keeping Drosophila stocks alive is labour-intensive, so stock centres routinely assess which stocks they can afford to keep. During the mid-1990s, this aspect of collections management was made visible when the Genetics Society of America (GSA) established a panel of expert geneticists to assess the current state of Drosophila stock provision.^39^ The Mid-America Stock Center was due for an NSF review, and many researchers worried that the collection was no longer sufficiently relevant. The GSA hoped to offer pre-emptive advice on how the collection might be improved. The Mid-America collection had been founded by Irwin Oster in 1958, and comprised in large part duplicates of stocks kept by pre-eminent geneticist Hermann Muller at the University of Indiana.^40^ Oster continued caring for this collection after Muller died, moving with it to Bowling Green State University in the late 1960s. While Oster initially oversaw the collection, he suffered from ill health and his wife Phyllis took responsibility for the collection’s day-to-day maintenance, while another researcher in the department oversaw its running. In contrast to the Species Stock Center, which had faced challenges in adequately caring for its stocks, the Mid-America Stock Center received praise from the GSA panel for the exceptional care given by Phyllis Oster and the excellent condition of flies. The Mid-America Stock Center was understood by many to be an invaluable resource for Drosophila research, providing stocks to colleges and high schools for teaching purposes as well as to researchers. But the panel was concerned that the centre was not keeping up with the ‘needs of the research community’. They claimed that the collection contained too many ‘archival’ stocks and commented (critically) that the stock list in the mid-1990s bore ‘a striking resemblance’ to Muller’s original holdings.^41^ This group used the term ‘archival’ to mean old or historic stocks, no longer useful for research. The Mid-America collection had – in the opinion of some – not been kept up to date, which was cited as a significant reason for its closure.

The same GSA panel had no such worries about the BDSC collection, which it judged to be ‘in excellent shape’.^42^ BDSC collections manager Kathleen Matthews had been overseeing its composition for a decade, since geneticist Thomas Kaufman had first brought the collection to Indiana from Caltech in the late 1980s.^43^ Matthews had been an experienced postdoc when she agreed to manage the collection, a job that she initially took on alongside her own research.^44^ After the Mid-America Stock Center closed in 1997, many of its stocks were moved to Bloomington. Ramping up the BDSC workforce and facilities to cope with these new accessions, Matthews chose to hire new staff scientist Kevin Cook. With a masters and PhD in biology, Cook came to share the management responsibilities of the centre with Matthews. Sometimes called ‘curators’ and sometimes ‘collections managers’, both scientists noted that they ‘go back and forth on what to call ourselves’. Alluding to the relative invisibility of their role, they also remarked that collections managers and curators ‘don’t always fit neatly into the structure of things’, ‘the structure’ being that of the local biology department and its faculty.^45^ The BDSC managers are responsible for the ongoing relevance of the collection.

Why do managers keep some stocks and discard others? What are the ‘needs of the research community’ and how are they judged? With thousands of Drosophila labs worldwide, ‘the community’ is an abstract concept that is highly flexible as to whom it includes, and which varies in relation to time and place. Historically, particular stock centres have been connected to powerful researchers and laboratories: at Caltech (Thomas Hunt Morgan, Ed Lewis), Cold Spring Harbour (Calvin Bridges, Milislav Demerec) and Bloomington (Hermann Muller). The GSA panel believed that to maintain the relevance of a collection, Drosophila stock centres had to be attached to active research labs. When the GSA panel judged that the Mid-America Stock Center was failing to meet the ‘needs of the community’, it was in part owing to the perception that there were not enough Drosophila researchers local to it.^46^ The panel reported that it felt that the university did ‘not have a CRITICAL MASS of “state-of-the-art” Drosophila research laboratories’. It recommended that the stock centre employ a full-time curator with a PhD in Drosophila genetics ‘with a sophisticated understanding of the needs of the research community and a commitment to revamp the center to meet those needs’.^47^ This was another register of perceived ‘relevance’ – the GSA panel believed that the composition of a collection would be strong with a strong institutional connection to cutting-edge research.

The European Drosophila Stock Centre, based in the northern Swedish town of Umeå, actively maintained its perceived relevance by brokering relationships with high-profile researchers. Officially founded in 1979 with funding from the Swedish Natural Science Research Council (Naturvetenskapliga forskningsrådet – NFR), its directors maintained informal relationships with high-profile researchers. One of those was British geneticist Michael Ashburner, who strongly supported this European venture and lobbied various funding bodies for its continuation. A later Umeå director, Åsa Rasmuson-Lestander, recalled Ashburner’s remarkable energy for establishing and supporting community resources: ‘we called him … to see how we should develop the stock centre: what we should discard, and what we should aim at’.^48^ For the Umeå centre, these relationships were essential, but in the end not sufficient. When the centre lost its European funding one reason cited was that Drosophila genetics was not sufficiently strongly represented in its host department.^49^

BDSC workers talk about the delicate curatorial work of de-accessioning stocks to maintain a functional collection. That institution expanded rapidly from the late 1980s – from around seven thousand stocks to over seventy thousand – in part because the centre began accessioning collections of lines produced by researchers in the course of large-scale systematic genetic ‘screens’.^50^ During the 1970s and 1980s, Drosophila became involved increasingly in the study of molecular development, in part owing to projects in which scientists induced mutations in large numbers (sometimes many thousands) of lines and screened those lines for interesting developmental phenotypes.^51^ These resulted in some striking discoveries, such as the highly conserved Toll developmental pathway and *Hox* patterning genes, which positioned Drosophila as an important model for biomedical research.^52^ Such screening projects still have an important place in Drosophila genetics, though screens have become more precise (producing targeted mutations) and involve techniques for allowing researchers to clone portions of the Drosophila genome.^53^ A screen has the potential to create a set of tools (fly lines) with ongoing uses, and can help position a laboratory or institution at the centre of a new subfield. But funding bodies will typically only provide money for such projects if resulting stocks can be made available to the rest of the community. Most labs do not have the resources to maintain so many stocks (or the means to study such a large wealth of material), so making such resources available for others to use is possible only if a stock centre agrees to accession them.

Stock centres are often keen to accession novel collections of stocks, but screens can produce massive numbers of new lines (these can be in the region of 150,000), far beyond the capability of a stock centre.^54^ Matthews, Cook and their fellow curators have become cautious about accepting uncharacterized lines whose utility is not clear, and have tried to be ruthless when it comes to getting rid of flies from older screens that are no longer useful. They gauge how interesting or useful certain mutants are by monitoring how many papers they have yielded, and by consulting stock donors and users. But such negotiations can still cause tensions. In some instances, donors have become sentimentally attached to stocks, especially if vast amounts of work and money went into making them.^55^

The BDSC helps to maintain the relevance of its collection via a Scientific Advisory Board, which advises on ‘policy issues including acquisitions and deaccessions, coordination with other collections, cost recovery and community interactions’.^56^ The centre also consults users through its webpages. Approximately once a year the BDSC carries out a de-accessioning protocol via a webpage dedicated to ‘pruning’ – terminology that chimes with the agriculturally inflected ‘culturing’ and ‘feeding’ of other aspects of BDSC activities. The ‘pruning’ webpage lists ‘selected obsolete, redundant or low-use stocks’ as candidates for removal.^57^ By publicizing this list – which in June 2017 comprised 1,192 stocks – the centre gives researchers the opportunity to contest the candidates for de-accession, or to order the stocks themselves so that they can be kept locally.^58^ Collections manager Cook commented that it could be hard and time-consuming to get rid of stocks. Director Kaufman notes cheerfully that the 1,500-strong collection that he moved from Caltech is barely recognizable within the holdings of what they hold today.^59^

This emphasis on de-accessioning for a collection to ‘remain relevant’ marks a sharp contrast with some other kinds of biological collection. Many repositories – e.g. of seeds, tissues and blood – are assembled to mitigate loss of diversity. The loss of particular specimens within collections of those kinds has the potential to damage the composition and value of the collection as a whole. Living Drosophila stock centres are different: no one wants to lose scientifically valuable lines, but the destruction of specific stocks is sometimes required to maintain relevance to the communities that they serve and therefore the credibility of a collection.^60^ Moreover, unlike tissues or blood or wild strains, transgenic Drosophila can (in principle) be made again. Without a vigilant commitment to de-accessioning objects a biological stock centre might end.

## Documentation

The Mid-America Stock Centre also struggled to keep up with changing genetic nomenclatures and documentation formats. A crucial aspect of stock-centre work is the regulation of the relationship between a stock, its label and its references in a database. With a new accession, managers assess how it will be entered into its catalogue, sometimes changing its name so that it conforms to nomenclatural standards.^61^ Historically, local stock lists defined the identities of stocks, in particular their genetic identities, their provenance and the publications resulting from experiments carried out on them. Since the 1990s, community databases – FlyBase in the case of Drosophila research – have made commensurate the documentation of different stock centres.^62^ Databases are relied upon by researchers to maintain a chain of reference between organism, genetic construct and experiment.^63^ For two institutions – the BDSC and the Mid-America Stock Center – this kind of documentary maintenance work determined which institution would succeed and which would end.

Database curators are crucial for data-intensive biology today: they mediate and maintain community cohesion, and define who can talk to and collaborate with whom.^64^ Nomenclatures are one of the most fiercely contested areas of negotiation for any geneticist, journal editor, collections manager or database curator. For over a century, large-scale genetic-mapping projects have relied on community collaboration, and therefore the shared understandings of names and symbols. The circulation of nomenclatures in part defines communities.^65^ At the same time, nomenclatures are notoriously hard to standardize. Researchers are often doggedly attached to the symbols they are used to, which testifies to their importance and specificity for their users. As methods, questions and genetic technologies change it can be hard to find new standards that adequately serve everyone.^66^ One quip – attributed to Kaufman and quoted to me by several interviewees – is that ‘geneticists would rather share a toothbrush than a nomenclature’.^67^ But Drosophila are small and unremarkable: without labels or documentation, and the means to communicate that information, a stock is functionally useless. To mitigate the tendency for nomenclature proliferation, the circulation of flies within a community of researchers relies on technologies for ordering, circulating and comparing standardized stock lists.

For more than eighty years, the maintenance and distribution of living mutant organisms for genetic research have been intimately connected to technologies for managing and communicating information about them. In the 1940s, the National Research Council (NRC) convened a meeting on biological stock collections to sketch guiding principles for the provision of standardized organisms. Delegates agreed that the most effective stock-keeping infrastructures belonged to the maize and Drosophila research communities. What made these exceptional was the newsletters they had established for distributing up-to-date information about mutants, new findings and the location of stocks.^68^ The *Maize Genetics Cooperation News Letter* had been started by Rollins Emerson at Cornell University in 1932, and soon after the *Drosophila Information Service* (*DIS*) was established by Milislav Demerec at Cold Spring Harbor.^69^ Other genetic-research communities – those studying dogs, rats, mice, fish, monkeys, cats, guinea pigs, rabbits, corn, chickens, amphibians and fungi – had far patchier provision, relying on individual labs, bird fanciers, pharmaceutical companies and agricultural breeders, and none had newsletters. For these researchers, reliable, standardized information about reliable, standardized research organisms was hard to come by. Delegates of the 1940 meeting agreed that the care and distribution of mutant organisms went hand in hand with technologies for distributing information about them – at this time, principally newsletters and the postal service.

*DIS* functioned in part to demarcate areas of research and to communicate preliminary results. But its most urgent purpose was to circulate information about which labs possessed which fly stocks, and how researchers could obtain them.^70^ Indeed, Drosophila researchers were only able to obtain copies of *DIS* if they allowed the newsletter to share their stock lists. So *DIS* promoted community norms by permitting and regulating the sharing of flies, and in so doing adjudicated membership of the Drosophila community.^71^ *DIS* was understood to be so important to the efficient maintenance and circulation of stocks that when Demerec retired in 1960 it was the NRC Committee on the Maintenance of Genetic Stocks that assumed editorship and ensured that the newsletter would continue for the next thirty years.

Another important printed technology used by geneticists was the Drosophila mutant catalogue, first published in 1944 under the title *The Mutants of Drosophila Melanogaster* by Calvin Bridges and Katherine Brehme. It ordered all laboratory mutants according to allele and gave descriptions of their phenotypes and genetic information. The book was revised in the 1950s (by Brehme), in the 1960s (by Dan Lindsley and Ed Grell of Oak Ridge National Laboratory) and in the late 1980s by Lindsley and Georgiana Zimm – these last two editions are known collectively and colloquially as ‘the Red Book’.^72^ Paper-based infrastructure (stenographic labour, postal services, printing technologies) and women’s editorial, inscription and intellectual labour were essential for harnessing and maintaining the reproductive labour of research organisms.

*DIS* remained the main means for circulating stock information, and the Red Book a principal reference for mutants until the early 1990s. Then, several powerful members of the Drosophila community garnered funding from National Institutes for Health (NIH) to establish a cross-referenced database of gene mutants, chromosomal aberrations, bibliographies, laboratory address lists and resources for obtaining living stocks. FlyBase was one of the first model-organism databases and was run by four university research groups, at Cambridge, Harvard, Indiana and UCLA. First circulated on magnetic tape, and later on the World Wide Web, it was seen by many as an essential tool for the Drosophila community and the Human Genome Project.^73^ FlyBase developed in lockstep with the Bloomington stock centre. In 1991, BDSC founder Kaufman also became a founding principal investigator for FlyBase, and Matthews deeply involved in its planning and organization. Indeed, before FlyBase was established Matthews herself had embarked on one of the earliest attempts to put information about Drosophila mutants onto a database, producing floppy discs containing the Bloomington stock lists, which could be sent through the post. The database developed by Matthews became one of the resources cited in early grant proposals for FlyBase.^74^ Owing to Kaufman’s involvement, Bloomington became one of the four sites to run FlyBase, and there the FlyBase team shared a floor with BDSC workers. Reflecting back on this period, Matthews felt that these efforts to ‘attach information to things’ (flies) had been crucial for the efficient running of the BDSC.^75^ This intimate institutional connection – managed by Kaufman and Matthews – meant that the Bloomington stock lists were quickly transposed into a format that was compatible with the new FlyBase software. These lists became one kind of cross-referenced information entered into the FlyBase database.

FlyBase quickly became a well-used resource for Drosophila biologists, and stock centres beyond Bloomington had to keep up. In Europe, the principal stock keeper of the Umeå centre, Karin Ekström, recalled the labour-intensive work of assessing which stocks conformed to FlyBase rules, and changing those that did not. To do this, Ekström elicited help from Cambridge FlyBase computer software engineer Aubrey de Grey, and recalls having to subsequently decipher and correct the names on handwritten vial labels.^76^ This administrative work was challenging and time-consuming, but the Umeå stock centre successfully transitioned to the new formats. Meanwhile, the Mid-America Stock Center struggled. An oft-repeated complaint about the centre, noted by the GSA panel, was that it used an inconsistent nomenclature in its database of stocks, and that its managers had been slow to convert its stock lists to FlyBase standard usage. It noted that while the ‘Bloomington list has now been made to conform to FlyBase usage … this has not yet occurred for Bowling Green’. It reiterated the labour required to bring the stock lists up to date: the ‘Mid-America stock lists are far from the FlyBase standard, and it will take a lot of work to achieve this standard’.^77^ The report made clear that the incompatibility of the Mid-America stock lists with the FlyBase database was putting the whole collection in jeopardy. By contrast, the tight coupling of FlyBase with the BDSC positioned the latter as the pre-eminent collection of living Drosophila stocks. FlyBase represented a new documentation technology for fruit fly genetics, and its practices affected the dynamics of authority of the stock centres.

Today, the BDSC and FlyBase actively collaborate with researchers to set standards for new nomenclatures. When BDSC workers plan to accession a large array of flies – from, say, a new kind of genetic screen – they contact FlyBase curators to discuss symbols. Sometimes this can happen before a paper is published: the lab responsible for a new collection might contact the BDSC as it writes up its results. In this case, the stock centre might establish a three-way discussion – with the relevant laboratory scientists and with FlyBase curators – to negotiate the most suitable nomenclatures for the new genetic constructs. Different stakeholders might have different requirements. Researchers may wish to adhere to local conventions, while FlyBase may want a system of naming that will fit existing rules for similar kinds of genetic construct and that can be used by other scientists in the future. Those at the BDSC have additional pragmatic considerations: they want to use ‘valid FlyBase symbols’ but need to be able to write these onto labels and vials. Describing these negotiations, one FlyBase curator put it, ‘[we need to] know [that the nomenclature is] going to work for everyone: Bloomington doesn’t want the symbols to be too long, and its got to fit in [FlyBase] rules’.^78^ Collections managers must maintain links with database curators and actively negotiate the identities and documentation of stocks so that they can be accessed and valued.

## Money

In the late 1990s the Umeå stock centre closed, owing to a lack of money. The problem of how to secure stock-collection funding had first become particularly visible in the late 1930s when Rockefeller Foundation director Warren Weaver raised with Vannevar Bush of the Carnegie Institution of Washington the possibility of funding a central institution for all kinds of biology stocks, including flies, mice, rabbits and fungi.^79^ Both philanthropic organizations funded multiple institutions for biological stock provision and Weaver was worried that such collections were proliferating unnecessarily. He wondered whether the Rockefeller and Carnegie could coordinate their funding, or even establish ‘a comprehensive centre for genetic stocks’. To gather the opinions of biologists he proposed a conference, to be convened by the NRC, to evaluate ‘the role of the government’ and the roles of ‘commercial supply services, of various university departments, museums, etc.’, in maintaining and distributing such stocks.^80^

The Conference on the Maintenance of Pure Genetic Strains took place in Washington in 1941 and brought together biologists with special interests in stock provision.^81^ Delegates shared their concerns about existing arrangements and felt that in most cases specialist, organism-specific stock centres were crucial. They ultimately rejected Weaver’s suggestion of a single institution to cater for all organisms, instead deciding that a heterogeneity of institutions was the safest organizational structure: ‘pure line strains could best be maintained by the workers most actively engaged in their study, whether in Government, university, museum or institutional laboratories’.^82^ But the coordination of stable funding remained a difficult problem, and to address it the NRC appointed a Committee on the Maintenance of Genetic Stocks, which would to oversee and coordinate provision between private, federal and commercial institutions.

The private philanthropic funding of stock centres started to change after the Second World War with the growth of US federal funding for science. In 1958, the NSF awarded a grant to Caltech stock centre for its collection, which was followed soon after by an award to a new stock centre in Philadelphia.^83^ By the 1980s, the three major US Drosophila stock centres were funded through the NSF, which had made a commitment to renewable funding for infrastructure. During that decade, though, NSF officials began to question the extent to which it was shouldering the burden of science infrastructure projects. They reasoned that by paying the full cost of stock provision the NSF was essentially subsidizing grants provided by other funding bodies, especially the richly funded NIH.^84^ NSF’s programme director in eukaryotic genetics, DeLill Nasser, calculated how much of the fly community resources the NSF and NIH were paying for, and by the mid-1990s was able to persuade the NIH that it needed to contribute to BDSC funding. Until this moment the Drosophila stock centres had not charged money for stocks – proposals to implement remuneration had been strongly resisted by some.^85^ But when the NSF and NIH began cooperative funding, they required the stock centre to implement a ‘cost recovery programme’, whereby labs would pay a small fee in exchange for a stock. This amount was initially voluntary, but not enough users offered funds, so from the late 1990s the stock centre began charging a small amount for each stock – a practice that remains in place today.^86^ At the BDSC today ‘cost-effectiveness’ is the principal guide for deciding which stocks to accession and which to jettison. Their break-even point is about six orders a year for a stock, so heavily used stocks subsidize those that get ordered less. The collections managers explained: ‘we don’t expect every stock to pay for itself, but if we get too many stocks that don’t begin to pay for themselves, then … the system will collapse’.^87^

The Umeå stock centre struggled with another form of voluntary subscription. The centre first received state funding for stock provision in 1979 when the Swedish National Science Foundation agreed support, convinced that it would put Umeå – and Swedish biology generally – on the European stage. Sure enough, in 1981, the centre was ‘adopted’ by the European Science Research Councils, which permitted the centre to obtain part of its funding through subscriptions from the research councils of member states.^88^ The 1970s and 1980s were marked by a decisive flourishing of Drosophila genetics in Europe and elsewhere. But Umeå asked research councils only for voluntary subscriptions, and although several did pay up the stock centre found it impossible to force recalcitrant research councils to contribute. Germany was a surprising culprit, one stock centre director remembers, because genetic research was thriving and German labs were supportive of the centre.^89^ Then, to the dismay of many biologists in Europe, the mid-1990s saw a decisive change to the European Commission’s policy on science funding. The commission funded science and technology through five-year ‘framework programmes’, with a budget of billions of euros. The fourth framework programme (1994–1998) had made an explicit commitment to funding scientific infrastructures ‘that are beyond the means and competence of national authorities and private initiative’, including stock centres.^90^ However, for the fifth framework programme (1998–2002), the European Commission ruled that operational costs for infrastructure would not be supported.^91^ Umeå tried to charge laboratories per stock but found that bank charges were too high to manage so many different currencies. The labour of processing these payments fell on the senior stock keeper at the centre and became too timeconsuming to keep up. In the year 2000 the European Drosophila Stock Centre closed, sending most of its stocks to a new centre in Kyoto.^92^

**Figure 3:**
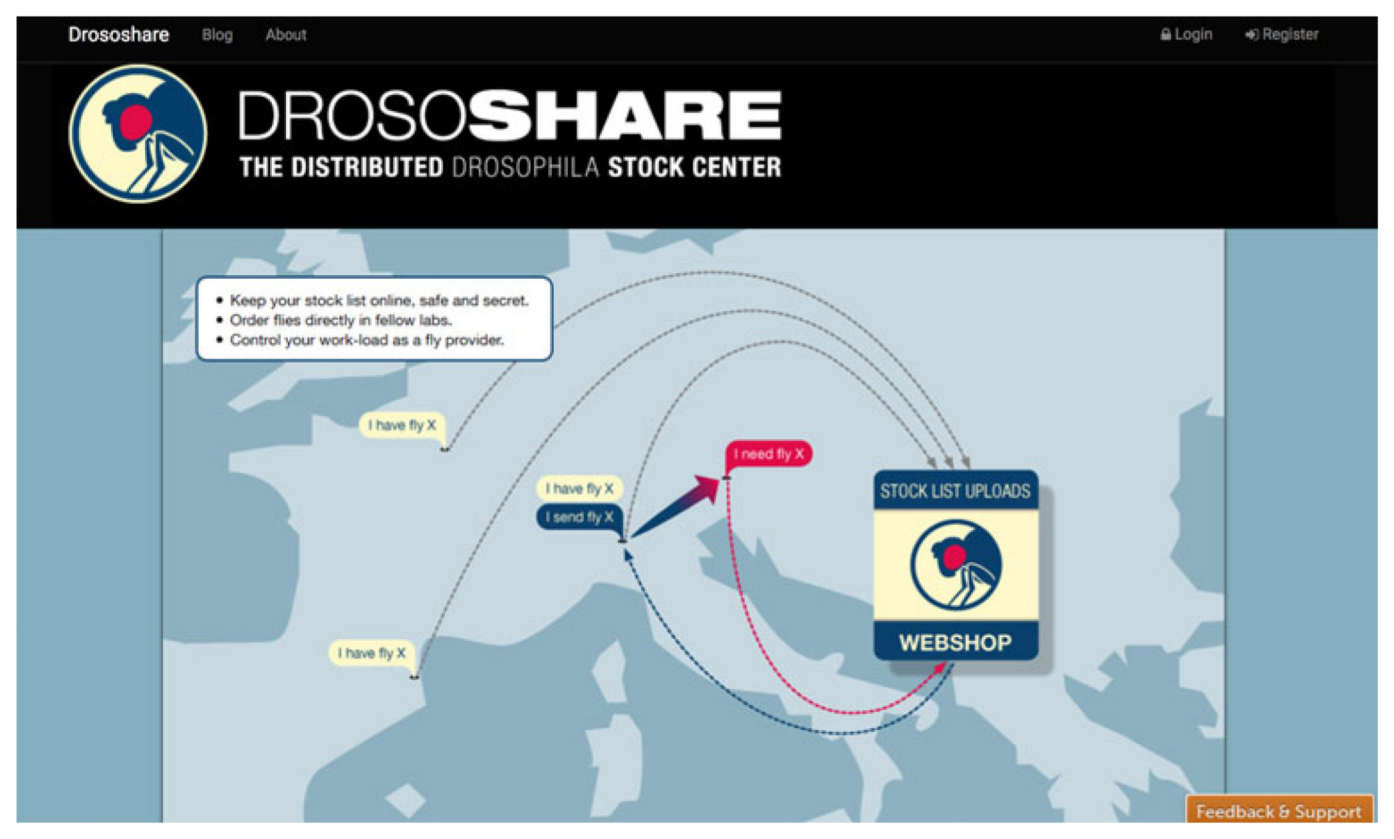
A screenshot of the Drososhare landing page. Logo and design by Daniel Wagner in collaboration with Julien Columb (www.drososhare.net, accessed 5 August 2017). Reproduced under CC BY 4.0, http://doi.org/10.5281/zenodo.3373817.

In an intriguing twist to the century-long history of managing Drosophila stocks and information about them, a new initiative for the community was announced in August 2017. The Drososhare website had the tagline ‘The Distributed Drosophila Stock Center’ and was an initiative based in Berlin that combined data-management software with a social-media aesthetic to offer a new service to labs (Figure 3). Its advertisements, which it circulated via Twitter, entreated labs to ‘send us your stock list and we will: clean stock names in your list ($2/stock), [and] integrate clean stocks into FlyBase (free)’. With these ‘cleaned’ lists, the platform promised to facilitate the movement of flies between labs (Figure 3). Although the group charged dollars for standardizing names and symbols attached to the stocks (‘cleaning’), Drososhare also proposed a system of debit and credit, whereby fly providers would ‘get bonus points (drosocoins) for each fly sharing’, which could then be ‘spent’ on requests for flies.^93^ The phrase ‘distributed stock centre’ was, of course, not quite accurate: Drososhare did not keep stocks, but sought to make and regulate exchanges between highly dispersed labs. It neatly cohered with the aesthetics and technologies of ‘the sharing economy’ – a phrase that refers to a range of commercial companies that use Internet technology to mediate distributed freelance employment. In this sense, Drososhare was less like a stock centre and more like the newsletter *DIS*, which as well as being a source of news, informal results and address lists, had also circulated information about who had what stocks and how they might be requested. However, *DIS* had been able to actively adjudicate community membership: a laboratory could only subscribe to *DIS* if it also shared its stock lists with the newsletter. Drososhare was a new initiative that did not offer additional community resources, so it had no such leverage.

Many believed that the initiative was overdue – after all, Drosophila researchers’ commitments to ideas of ‘sharing’ and ‘community’ have been central to the identity of the field for much of its history. However, by November 2018, Drososhare had closed. As well as difficulties in recruiting participants, those working for conventional stock centres had had reservations about this model of Drosophila provision. One question was whether an organization such as Drososhare could sufficiently standardize stock lists for the efficient circulation of flies. Another was that Drososhare might circulate flies derived from lines otherwise kept at stock centres, thereby potentially depriving those stock centres of income (in some ways analogous to the potential threats to hotels and taxi services posed by organizations like AirBnB and Uber). The Drososhare initiative certainly had the potential to facilitate the circulation of flies, and to alter the range and representation of stocks in labs and stock centres, but some believed it also threatened the continued existence of stock centres in and of themselves.

## Conclusion: ‘libraries’ and ‘archives’

All in all, the late 1990s were precarious for Drosophila stock centres. Funding from several sources was shrinking just as the expansion of genetic screens was dramatically amplifying stock numbers. Federal funding bodies in the US and Europe were renegotiating who should pay the operational costs of infrastructure. FlyBase was moving onto the World Wide Web and quickly becoming a new and dominant authority on communication standards. Notwithstanding the establishment of a new major stock centre in Kyoto in 2001, the result was the concentration of US and European *D. melanogaster* stocks at a single centre (the BDSC). In part owing to its institutional connection to FlyBase, BDSC workers were able to maintain scientific relevance, effectively document and communicate their holdings, and offer standards for how these should be done.

If Drosophila stock centres are collections that require curation, care and catalogues, how are they similar to and distinct from museum collections, archives, seed banks or tissue repositories? In 1993 the NSF brought together curators of living-stock collections across the biological sciences to discuss their future funding and management. Matthews of the BDSC co-authored a report of this meeting in the magazine *National Genetic Resources Program* [NGRP] *News*. In explaining the importance of living-stock collections to science, the authors drew a distinction between biological ‘germplasm’ collections, which they characterized as ‘archives’, and genetic-stock centres, which they described as ‘libraries’.^94^ The term ‘archive’ in this formulation refers to collections that are intended to be permanent and complete, such as tissues,^95^ seeds^96^ and animals,^97^ assembled to mitigate predicted loss of diversity in response to imagined scenarios years in the future.^98^ Collections of these kinds share features of museums and archives: in the language of the present issue, they are ‘unique’ collections: each object is valued for its unique historical (or geographical, or genetic) characteristics.

Like libraries, stock centres are ‘working’ collections (see Jardine, Kowal and Bangham, this issue). They perpetually reproduce lines of flies that are valued for their distinctive genetic features, and which could (in theory) be replaced by another stock with the same genetic characteristics – either a duplicate or (in some cases) another remade from other lines. In stock centres, one copy can be substituted for any other: precisely echoing the choice of the term ‘library’ in the 1993 National Research Council report. Stock centres and libraries are routine resources: they are dynamic, emphasize utility and are valued in relation to the immediate laboratory needs of researchers. Their objects have the potential to be highly distributed – circulating far beyond the institution that cares for them. Stocks and library books might be added or discarded from a collection according to the fashions, preferences and technologies of their users.

Although this is a useful analytic distinction, the varied curatorial practices at Drosophila stock centres suggest that working and unique collections sometimes have much in common. Museums, germplasm repositories and archives all demand and create specialist labour, day-to-day care, appraisal, documentation and sustained funding. Archivists have been particularly articulate in recent accounts of the dynamic practices of appraisal and reappraisal that must be carried out to maintain an archive’s value in the face of fiscal pressures and of competing demands of scholars and other stakeholders.^99^ Perhaps one distinct feature of biological stock centres is that they necessarily make those dynamic processes visible, communicating with their users through websites or via selected scientist representatives. A second is that they are not just collections, but places of mass production: Drosophila are animals that reproduce themselves at an astonishing rate, and in striking contrast to books in libraries – or indeed the objects in frozen zoos, germplasm collections and museum collections – the stocks that circulate are copies of pure breeding lines. (Re)production and its management are key to the continuation of stock collections and their distribution to new laboratories.

Collections of living fruit flies constitute a distinct ‘layer’ to the history of Drosophila genetics (see Jardine, Kowal and Bangham, this issue), a layer comprising not just living animals but also the apparatus, people and practices that keep them alive. Its composition is negotiated by collections managers in collaboration with users, funders and database curators, who determine and stabilize the stocks available to science. This attention to care and maintenance leads to a broader point about the temporalities of Drosophila genetics and the significance of care.^100^ It reminds us that much Drosophila research deals with stocks inherited from past experiments or generations, so for many involved in Drosophila genetics, maintenance and care come first, prior to the design and production of novel genetic constructs. This also points to the significance of institutions: when living stocks exist as collections, they routinize and institutionalize curation and care. The BDSC remains the largest and most authoritative collection of laboratory fruit flies in the world, just like its institutional antecedents, at Caltech and Columbia University. En masse, laboratory Drosophila have stabilized protocols of care that outlive individual biologists.

## Figures and Tables

**Figure 1:**
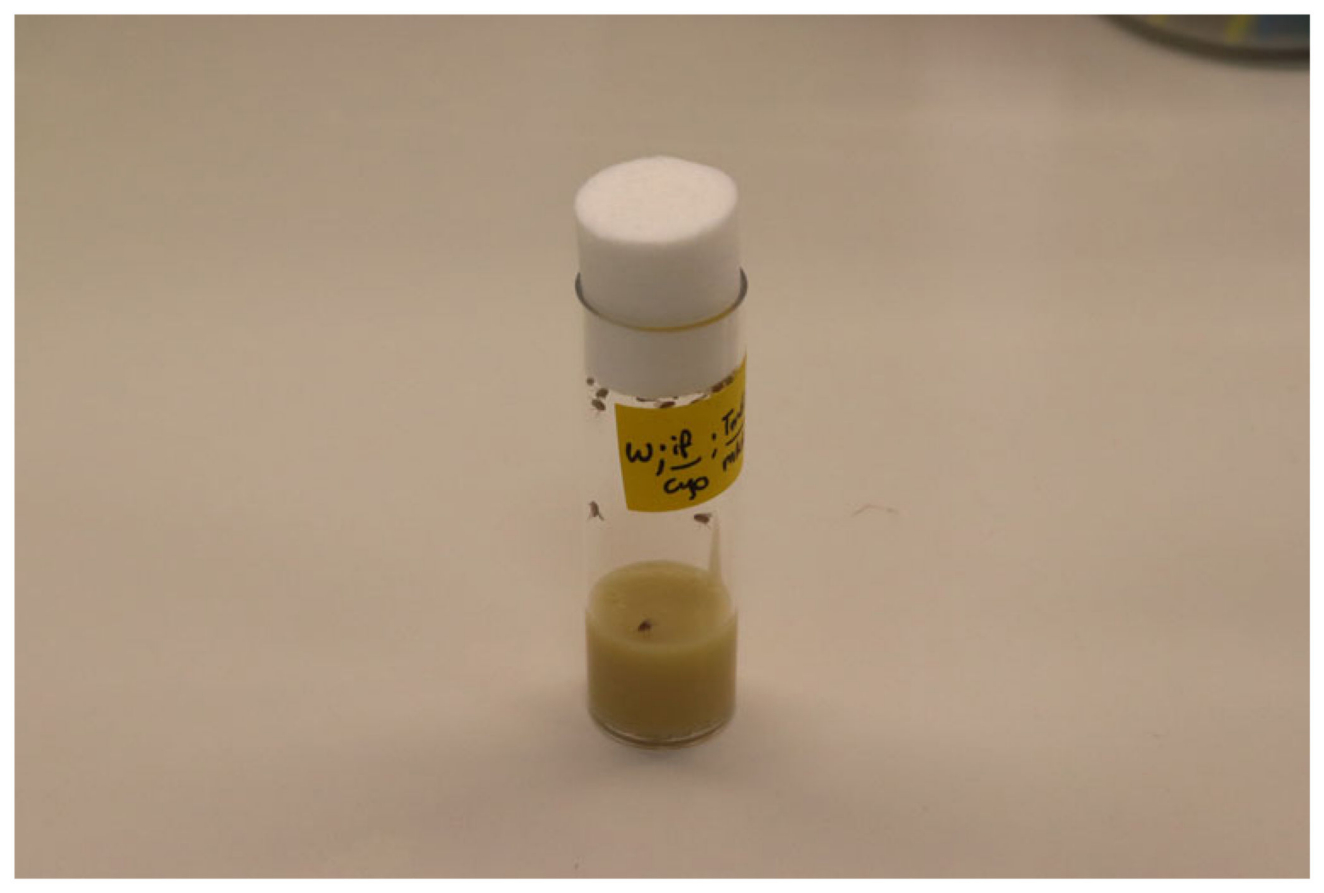
*Drosophila melanogaster* flies in a vial containing yeast-agar food, labelled with sticky tape and felt tip pen. Photograph by the author.

## Footnotes

1 Nadia Rosenthal and Michael Ashburner, ‘Taking stock of our models: the function and future of stock centres’, *Nature Reviews Genetics* (2002) 3, pp. 711–717.

2 For a sense of the astonishing arrays of living scientific collections see ‘Culture collections information worldwide’, at www.wfcc.info/index.php/collections/display, accessed 1 April 2018; Robert L. Jarrett (ed.), *The Biological Resources of Model Organisms*, London: Taylor & Francis Ltd/CRC Press, 2019.

3 See www.oed.com/view/Entry/190595, accessed 17 October 2017.

4
*Drosophila* means ‘dew-loving’, and *melanogaster* ‘black bellied’. *Drosophila melanogaster* is the species most commonly used for genetic research and is also known as the ‘vinegar fly’ or ‘wine fly’ for its attraction to fermenting fruit.

5 The ease with which Drosophila move through worldwide postal systems varies across countries and continents and has a complex history.

6 Michael E. Lynch, ‘Sacrifice and the transformation of the animal body into a scientific object: laboratory culture and ritual practice in the neurosciences’, *Social Studies of Science* (1988) 18, pp. 265–289; Robert E. Kohler, *Lords of the Fly: Drosophila Genetics and the Experimental Life*, Chicago: The University of Chicago Press, 1994; Donna Haraway, *Modest_Witness@Second_Millenium.FemaleMan(C)_Meets_OncoMouseTM*, New York: Routledge, 1997.

7 Some strains contain the morphologically anomalous ‘attached-X’ and ‘balancer’ chromosomes (and other variants), used as technologies to maintain mutations stably in place, generation after generation.

8 From the 1980s transgenic flies were created using ‘P-elements’ (a naturally occurring piece of DNA that can pop in and out of fly genomes) to introduce specific mutations or gene deletions. Newer techniques allow researchers to insert constructs into highly specific areas of the genome, most recently using CRISPR.

9 Kohler, op. cit. (6); Karen A. Rader, *Making Mice: Standardizing Animals for American Biological Research, 1900*–*1955*, Princeton, NJ and Oxford: Princeton University Press, 2004; Lynda Birke, Arnold Arluke and Mike Michael, *The Sacri*fi*ce: How Scienti*fi*c Experiments Transform Animals and People*, West Lafayette: Purdue University Press, 2007.

10 Kohler, op. cit. (6).

11 The Caltech lab was run by Thomas Hunt Morgan; ‘curator of stocks’ was ‘Eleanor Nichols (Mrs F.K. Skoog)’, *Drosophila Information Service* (1934) 1, p. 68.

12 Michael R. Dietrich and Brandi H. Tambasco, ‘Beyond the boss and the boys: women and the division of labor in *Drosophila* genetics in the United States, 1934–1970’, *Journal of the History of Biology* (2007) 40, pp. 509–528.

13 Edward B. Lewis, ‘Drosophila stock center’, *Drosophila Information Service* (1958) 32, pp. 176–177. A year earlier, Irwin Herskowitz had used the term ‘stock center’ in a controversial proposal for a dedicated ‘drosophila research stock center’, which was not established: Irwin H. Herskowitz, ‘Proposed Drosophila research stock center’, *Drosophila Information Service* (1957) 31, pp. 181–182.

14 The exchange practices for other model organisms are similarly rich and varied: since the 1930s inbred mice have been exchanged for money, while worms and seeds (for example) have typically (but not always) been exchanged between labs for free. E.g. Rader, op. cit. (9); Hippokratis Kiaris, ‘Support for living stock collections: a mammalian stock center perspective’, *Trends in Genetics* (2019) 35, pp. 173–174; Sabina Leonelli, ‘Weed for thought: using Arabidopsis thaliana to understand plant biology’, PhD thesis, Vrije Universiteit, Amsterdam, 2007; Laura Pottinger, ‘“It feels connected in so many ways”: circulating seeds and sharing garden produce’, in Anthony Ince and Sarah Marie Hall (eds.), *Sharing Economies in Times of Crisis: Practices, Politics and Possibilities*, New York: Routledge, 2017, pp. 125–141.

15 The other major Drosophila stock centre in the world today is the Kyoto Stock Center, founded in 2001.

16 I draw on a rich recent sociological and historical scholarship on the routine care of experimental mice and other mammals: Nicole Nelson, ‘Model homes for model organisms: intersections of animal welfare and behavioral neuroscience around the environment of the laboratory mouse’, *BioSocieties* (2016) 11, pp. 46–66; Carrie Friese, ‘Realizing potential in translational medicine: the uncanny emergence of care as science’, *Current Anthropology* (2013) 54, S129–138; Gail Davies, Beth Greenhough, Pru Hobson-West and Robert G.W. Kirk, ‘Science, culture, and care in laboratory animal research: interdisciplinary perspectives on the history and future of the 3Rs’, *Science, Technology, & Human Values* (2018) 43, pp. 603–621; Robert G.W. Kirk, ‘Recovering the principles of humane experimental technique: the 3Rs and the human essence of animal research’, *Science, Technology, & Human Values* (2017) 43, pp. 622–648; Robert G.W. Kirk, ‘Care in the cage: materializing moral economies of animal care in the biomedical sciences, *c.*1945–’, in K. Bjørkdahl and T. Druglitrø (eds.) *Animal Housing and Human*–*Animal Relations: Politics, Practices and Infrastructures*, Oxford and New York: Routledge, 2016, pp. 167–184; Donna Haraway, *When Species Meet*, Minneapolis: University of Minnesota Press, 2008. For scholarship on practices of care (and the people who care) in biology beyond animals, Xan Chacko, ‘Creative practices of care: the subjectivity, agency and affective labor of preparing seeds for long-term banking’, *Culture, Agriculture, Food and Environment*, forthcoming; Karen Ingeborg Jent, ‘Making stem cell niches: an ethnography of regenerative medicine in Scotland and the United States’, PhD thesis, University of Cambridge, 2017.

17 A waxy coating on the surface of Drosophila embryos prevents water loss so freezing causes the catastrophic formation of ice crystals. Several announcements of freezing techniques have not yet yielded realistic protocols for routine stock keeping.

18 Glass vials are autoclaved, but plastic vials are typically deep frozen to kill larvae and pupae, then thrown away with other laboratory garbage. In the late 1990s concern about the use and circulation of genetically modified organisms formalized requirements for Drosophila disposal. US labs and stock centres follow NIH guidelines on disposal: https://osp.od.nih.gov/wp-content/uploads/2013/06/NIH_Guidelines.pdf. For many working with Drosophila, ‘to autoclave’ is verbal shorthand for ending a stock.

19 For some histories of stock breeding, in agriculture and science, see Bonnie Tocher Clause, ‘The Wistar rat as a right choice: establishing mammalian standards and the ideal of a standardized mammal’, *Journal of the History of Biology* (1993) 26, pp. 329–349; Kathy J. Cooke, ‘From science to practice, or practice to science? Chickens and eggs in Raymond Pearl’s agricultural breeding research, 1907–1916’, *Isis* (1997) 88, pp. 62–86; Bert Theunissen, ‘Breeding for nobility or for production? Cultures of dairy cattle breeding in the Netherlands, 1945–1995’, *Isis* (2012) 103, pp. 278–309.

20 A stock centre might, for example, absorb a systematic screen into its collection, but maintain its identity as a distinctive set of genetic tools.

21 For the documents and catalogues defining a museum collection’s shifting ontologies see Porter, this issue.

22 On producing and maintaining reference in the collection of specimens see Bruno Latour, *Pandora*’*s Hope: Essays on the Reality of Science Studies*, Cambridge, MA: Harvard University Press, 1999, pp. 24–79.

23 The constant negotiation and management of names and the identities of genetic objects might be thought of as ‘ontological labour’. For an important set of such practices see the flourishing scholarship on ‘bioontologies’: Sabina Leonelli, ‘Documenting the emergence of bio-ontologies: or, why researching bioinformatics requires HPSSB’, *History and Philosophy of the Life Sciences* (2010) 32, pp. 105–125; Sabina Leonelli, Alexander D. Diehl, Karen R. Christie, Midori A. Harris and Jane Lomax, ‘How the gene ontology evolves’, *BMC Bioinformatics* (2011) 12(325), p. 1–7; Sabina Leonelli, *Data-Centric Biology: A Philosophical Study*, Chicago and London: The University of Chicago Press, 2016. For more on classification, naming and communication in biology, and particularly genetics, see Geoffrey C. Bowker and Susan Leigh Star, *Sorting Things Out*, Cambridge, MA: Harvard University Press, 1999; Paul N. Edwards, Matthew D. Mayernick, Archer L. Batcheller, Geoffrey C. Bowker and Christine L. Borgman, ‘Science friction: data, metadata, and collaboration’, *Social Studies of Science* (2011) 41, pp. 667–690; Brian Balmer, ‘Managing mapping in the Human Genome Project’, *Social Studies of Science* (1996) 26, pp. 531–573; Soraya de Chadarevian, ‘Mapping the worm’s genome: tools, networks, patronage,’ in Jean-Paul Gaudilliere and Hans-Jörg Rheinberger, eds., *From Molecular Genetics to Genomics: The Mapping Cultures of Twentieth Century Genetics*, Oxford and New York: Routledge, 2004, pp. 95–110; Stephen Hilgartner, ‘Making maps and making social order: governing American genome centres 1988–93’, in Gaudillière and Rheinberger, op. cit., pp. 113–128; Karen A. Rader, ‘“The mouse people”: murine genetics work at the Bussey Institution, 1909–1936’, *Journal of the History of Biology* (1998) 31, pp. 327–354.

24 Kohler, op. cit. (6); Christopher M. Kelty, ‘This is not an article: model organism newsletters and the question of “open science”’, *BioSocieties* (2012) 7, pp. 140–168; Rader, op. cit. (9); Sabina Leonelli, ‘Growing weed, producing knowledge: an epistemic history of *Arabidopsis thaliana*’, *History and Philosophy of the Life Sciences* (2007) 29, pp. 193–223; Leonelli, ‘Packaging small facts for re-use: databases in model organism biology’, in Peter Howlett and Mary S. Morgan (eds.), *How Well Do Facts Travel? The Dissemination of Reliable Knowledge*, Cambridge: Cambridge University Press, 2011, pp. 325–348; Sabina Leonelli and Rachel A. Ankeny, ‘Re-thinking organisms: the impact of databases on model organism biology’, *Studies in History and Philosophy of Biological and Biomedical Sciences* (2012) 43, pp. 29–36.

25 Leonelli and Ankeny, op. cit. (24).

26 [X] [X], ‘“stocks of simulans” [author and recipient retracted]’, 21 October 1996, papers held at BDSC, University of Indiana, Bloomington.

27 Taxonomists estimate that there are 1,600 species of Drosophila today. On the history of the study and classification of this genus see Patrick O’Grady and Rob DeSalle, ‘Phylogeny of the genus *Drosophila*’, *Genetics* (2018), 209, pp. 1–25.

28 The basic ingredients of *D. melanogaster* laboratory food have been stable for nearly a century, although different institutions use slightly different recipes for reasons (and with consequences) I will not go into here.

29 Anonymous, personal interview, 30 May 2017. For recipes and ingredients see the Species Stock Center webpage: http://blogs.cornell.edu/drosophila/recipes, accessed 20 December 2018.

30 Dietrich and Tambasco, op. cit. (12) pp. 524–525, note a remark by one former laboratory worker in a 1990s interview: ‘at least during my time [the 1940s and 1950s] – stock keeping just wasn’t to the taste of men, and women were more inclined to do this tedious, tiresome job without complaining’.

31 Drosophila are susceptible to infections by tiny white ‘mites’, which can make it hard for flies to thrive. Many institutions ‘quarantine’ new flies.

32 For a video of the robot see www.janelia.org/support-team/drosophila-resources, accessed 3 March 2019.

33 Anssi Saura, email to Jenny Bangham, 15 September 2016.

34 Anonymous, personal interview, 1 June 2017. On worry and care for facts and other technoscientific things see Maria Puig de la Bellacasa, ‘Matters of care in technoscience: assembling neglected things’, *Social Studies of Science* (2011) 41, pp. 85–106.

35 See https://bdsc.indiana.edu/about/personnel.html, accessed 15 June 2018.

36 Kevin Cook, email to Jenny Bangham, 15 June 2018.

37 [X] [X], [author and recipient retracted], 7 November 1996, papers held at BDSC, University of Indiana, Bloomington.

38 Anonymous, personal interview, 30 May 2017.

39 First established in 1931, the GSA organizes conferences and represents the interests of geneticists to funders. The GSA had a Drosophila Board – that is, an influential group of Drosophila researchers that represented the interests of fly geneticists to the GSA. In 1996 the Drosophila Board appointed the panel to evaluate stock centre provision.

40 Oster’s collection also included duplicates from Caltech collection, and an array of stocks from other labs. ‘*Drosophila melanogaster* stock centers’, *Drosophila Information Service* (1961) 35, pp. 107–108.

41 ‘Evaluation of the current status of the US public Drosophila melanogaster stock centers,’ 4 November 1995, revised December 1995, papers held at BDSC, University of Indiana, Bloomington, p. 3.

42 ‘Evaluation of the current status of the US public Drosophila melanogaster stock centers’, op. cit. (41), p. 4.

43 Kaufman has run a Drosophila research lab at Bloomington since the 1980s, and remains pivotal to the stock centre today.

44 ‘A remembrance: Dr. Kathleen A. Matthews and the Bloomington Drosophila Stock Center and FlyBase’, *Genes to Genomes* (blog), 9 April 2018, at http://genestogenomes.org/a-remembrance-dr-kathleen-a-matthews-the-bloomington-drosophila-stock-center-and-flybase, accessed 15 April 2018.

45 Both have faculty status within the University of Indiana, although both Matthews and Cook remarked that their positions are sometimes not recognized as such by those in the university community.

46 Ron Woodruff of the Bowling Green Department of Biological Sciences was a highly respected scientist, but the GSA panel noted that there was ‘no full-time experienced Drosophila researcher supervising the management of the stock center’. ‘Evaluation of the current status of the US public Drosophila melanogaster stock centers’, op. cit. (41), p. 5.

47 ‘Evaluation of the current status of the US public Drosophila melanogaster stock centers’, op. cit. (41), p. 5, original emphasis. The report concluded, ‘The bottom line is that the Mid-America stock center must make a dynamic and proactive effort to modernize its collection.’

48 Karin Ekström and Åsa Rasmuson-Lestander, personal interview, 5 December 2017.

49 Anssi Saura, personal interview, 29 January 2018.

50 This expansion was also due to the creation of systematic ‘deletion’ lines; that is, fly stocks missing overlapping small sections of their chromosomes, which are used for fine-scale genetic mapping.

51 Marcel Weber, ‘Walking on the chromosome: Drosophila and the molecularization of development’, in Gaudillière and Rheinberger, op. cit. (23), pp. 63–78; Weber, ‘Redesigning the fruit fly: the molecularization of Drosophila’, in Angela N.H. Creager, Elizabeth Lunbeck and Norton M. Wise (eds.), *Science without Laws: Model Systems, Cases, Exemplary Narratives*, Durham, NC: Duke University Press, 2007, pp. 23–45.

52 Some of this work resulted in a Nobel Prize (in 1995) for Drosophila geneticists Christiane Nüsslein-Vollhard (Germany) and Eric Wieschaus (US), which they shared with developmental geneticist (and former Caltech stock centre director) Edward B. Lewis.

53 Daniel St Johnston, ‘The art and design of genetic screens: *Drosophila melanogaster*’, *Nature Reviews Genetics* (2002) 3, pp. 176–188.

54 Rosenthal and Ashburner, op. cit. (1).

55 Matthews and Cook recalled the sometimes painful negotiations with scientists over lines that had outlived their usefulness: ‘They’d made those stocks themselves, and put a lot of … heart and soul into it, and they really didn’t want to see them autoclaved and they would have liked us to take them’. Kathleen Matthews and Kevin R. Cook, personal interview, 30 May 2017.

56 See https://bdsc.indiana.edu/about/advisory.html, accessed 20 December 2018.

57 See https://bdsc.indiana.edu/about/acquisition.html, accessed 20 December 2018.

58 The earliest federally funded stock centres employed a similar protocol: from the 1960s two NSF-funded Drosophila stock centres announced de-accessions in *DIS* at least one year before they were discarded.

59 Thomas Kaufman, personal interview, 11 February 2018.

60 Elsewhere in this issue, David Skinner usefully calls these ‘operational endings’.

61 For an insightful account of how an *Arabidopsis* stock centre describes and classifies new seeds arriving from donors see Leonelli, op. cit. (14), pp. 147–183.

62 Leonelli and Ankeny, op. cit. (24).

63 Latour, op. cit. (22).

64 Leonelli, *Data-Centric Biology*, op. cit. (23).

65 Sabina Leonelli, ‘Centralizing labels to distribute data: the regulatory role of genomic consortia’, in Paul Atkinson, Peter Glasner and Margaret Lock (eds.), *The Handbook of Genetics & Society: Mapping the New Genomic Era*, Oxford and New York: Routledge, 2009, pp. 469–486; Leonelli, *Data-Centric Biology*, op. cit. (23); Bowker and Star, op. cit. (23).

66 Jenny Bangham, ‘Writing, printing, speaking: rhesus blood-group genetics and nomenclatures in the midtwentieth century’, *BJHS* (2014) 47, 335–361.

67 Rachel Drysdale, personal interview, 21 January 2018.

68 ‘Conference on maintenance of pure genetic strains [summary report]’ (National Research Council, 27 January 1940), p. 4, Biology and Agriculture Division, Conf on Maintenance of Pure Genetic Strains, National Research Council, National Academy of Sciences, Washington.

69 Kelty, op. cit. (24).

70 Kohler, op. cit. (6); H.V. Wyatt, ‘The invisible made visible’, *Nature* (1987) 329, pp. 357–358.

71 Kelty, op. cit. (24).

72 The covers of the books were red. Brehme had a PhD in zoology and was supported by a fellowship from the Carnegie Institution of Washington at Cold Spring Harbor. Lindsley and Grell were aided by the editorial, indexing and typing work of Lucille Norton, Eileen Slaughter, Elizabeth VonHalle and Neva Hair.

73 ‘FlyBase: a Drosophila relational database’, *Human Genome News* (1992) 4(3), p. 2.

74 ‘The FlyBase research resource’ (grant proposal to NIH), 1991, papers held by FlyBase, Harvard.

75 Kathleen Matthews, personal interview, 30 May 2017.

76 Karin Ekström and Åsa Rasmuson-Lestander, personal interview, 5 December 2017.

77 ‘Evaluation of the current status of the US public Drosophila melanogaster stock centers’, op. cit. (41).

78 Anonymous, personal interview, 2 March 2018.

79 Warren Weaver, ‘To Ross Harrison’, 30 March 1939, Biology and Agriculture Division, Conf on Maintenance of Pure Genetic Strains: Proposed, National Academy of Sciences, National Research Council.

80 Weaver, op. cit. (79).

81 The animals discussed at the meeting included dogs, rats, mice, Drosophila, daphnia, fish, birds, monkeys, apes, cats, smaller rodents, guinea pigs, opossums, rabbits, corn, maize, chickens, amphibians, bacteria, fungi, moulds, protozoa.

82 ‘National Research Council, Division of Biology and Agriculture, Conference on Maintenance of Pure Genetic Strains, Revised Transcript of Discussion’ (National Research Council, 27 January 1940), Biology and Agriculture Division, Conf on Maintenance of Pure Genetic Strains, National Research Council.

83 Irwin I. Oster, ‘Establishment of a second Drosophila stock center’, *Drosophila Information Service* (1960) 34, pp. 123–124. Oster later moved his collection to Bowling Green, where it became the Mid-American Stock Centre. On NSF history see Toby A. Appel, *Shaping Biology: The National Science Foundation and American Biological Research, 1945*–*75*, Baltimore: Johns Hopkins University Press, 2000.

84 The NIH became a leading patron of biomedical research in the US after the Second World War. See e.g. Buhm Soon Park, ‘The development of the Intramural Research Program at the National Institutes of Health after World War II’, *Perspectives in Biology and Medicine* (2003) 46, pp. 383–402.

85 Irwin H. Herskowitz, ‘Proposed Drosophila research stock center’, *Drosophila Information Service* (1957) 31, pp. 181–182; Irwin I. Oster, ‘To Melvin Green’, 26 March 1958, papers held at BDSC, University of Indiana, Bloomington.

86 At the BDSC, first five stocks ordered in a year are seventeen dollars each. The next ninety-five are seven dollars each, and everything over a hundred are $3.50 apiece. Charges pay for 78 per cent of the BDSC’s operating costs. Disciplines, fields and communities have varied in their inclusion of money in the exchange of biological organisms; e.g. Rader, op. cit. (9), pp. 97–134.

87 Kathleen Matthews, personal interview, 30 May 2017.

88 Michael Ashburner, Åsa Rasmuson-Lestander and Anssi Saura, ‘The European Drosophila Centre’ (letter to European Colleagues), 8 March 1994, papers held by Anssi Saura.

89 Anssi Saura, personal interview, 29 January 2018.

90 F. Gros and G.P. Tocchini-Valentini, ‘In search of European excellence’, *Nature* (1994) 369, pp. 11–12.

91 This apparently took institutions like the European Bioinformatics Institute (EBI) and its parent institution, the European Molecular Biology Laboratory (EMBL), unawares, apparently especially because ‘research infrastructure’ had been a stated priority for FP5. Declan Butler, ‘Life science facilities in crisis as Brussels switches off funding’, *Nature* (1999) 402, p. 3; Michael Ashburner, ‘Europe must grant crucial funds for biological research’, *Nature* (1999) 402, p. 12.

92 The Kyoto Stock Center charges handling and postage fees, but its ongoing stock maintenance is supported by the Japan Agency for Medical Research and Development, and the Kyoto Institute of Technology. Toshiyuki Takano-Shimizu-Kouno and Takashi Ohsako, ‘Humanized flies and resources for cross-species study’, in Masamitsu Yamaguchi (ed.), *Drosophila Models for Human Diseases*, Singapore: Springer, 2018, pp. 277–288.

93 See https://drososhare.files.wordpress.com/2016/10/drososhare_flyer.pdf, accessed 20 October 2017.

94 Kathleen Matthews and Calvin O. Qualset, ‘Living culture collections a national asset: experts set recommendations for the future’, *NGRP Network News*, 1994.

95 Joanna Radin, *Life on Ice: The History of New Uses for Cold Blood*, Chicago: The University of Chicago Press, 2017.

96 Helen Anne Curry, ‘Breeding uniformity and banking diversity: the genescapes of industrial agriculture, 1935–1970’, *Global Environment* (2017) 10, pp. 83–113.

97 Carrie Friese, *Cloning Wild Life: Zoos, Captivity, and the Future of Endangered Animals*, New York: New York University Press, 2013; Joanna Radin, ‘Planning for the past: cryopreservation at the farm, zoo, and museum’, in Fernando Vidal and Nélia Dias (eds.), *Endangerment, Biodiversity and Culture*, Oxford and New York: Routledge, 2016, pp. 218–240; M. Chrulew, ‘Freezing the ark: the cryopolitics of endangered species preservation’, in Joanna Radin and Emma Kowal (eds.), *Cryopolitics: Frozen Life in a Melting World*, Boston, MA: MIT Press, 2017, pp. 283–305.

98 Radin and Kowal, op. cit. (97); Vidal and Dias, op. cit. (97).

99 Terry Cook, ‘The archive(s) is a foreign country: historians, archivists, and the changing archival landscape’, *American Archivist* (2011) 74, pp. 600–632.

100 Andrew L. Russell and Lee Vinsel, ‘After innovation, turn to maintenance’, *Technology and Culture* (2018) 59, pp. 1–25.

